# Single Cell Transcriptome Amplification with MALBAC

**DOI:** 10.1371/journal.pone.0120889

**Published:** 2015-03-30

**Authors:** Alec R. Chapman, Zi He, Sijia Lu, Jun Yong, Longzhi Tan, Fuchou Tang, X. Sunney Xie

**Affiliations:** 1 Department of Chemistry and Chemical Biology, Harvard University, Cambridge, MA, 02138, United States of America; 2 Graduate program in Biophysics, Harvard University, Cambridge, MA, 02138, United States of America; 3 Biodynamics Optical Imaging Center, School of Life Sciences, Peking University, Beijing, China; Seoul National University, REPUBLIC OF KOREA

## Abstract

Recently, Multiple Annealing and Looping-Based Amplification Cycles (MALBAC) has been developed for whole genome amplification of an individual cell, relying on quasilinear instead of exponential amplification to achieve high coverage. Here we adapt MALBAC for single-cell transcriptome amplification, which gives consistently high detection efficiency, accuracy and reproducibility. With this newly developed technique, we successfully amplified and sequenced single cells from 3 germ layers from mouse embryos in the early gastrulation stage, and examined the epithelial-mesenchymal transition (EMT) program among cells in the mesoderm layer on a single-cell level.

## Introduction

mRNA expression analyses have been extensively used in biomedical research by fluorescence in situ hybridization (FISH), qRT-PCR, and microarray, and recently have been carried out on the entire transcriptome with the advent of next-generation sequencing via RNA-seq[1]. In general, FISH at single molecule resolution[2–4] gives the most quantitative measurement, but has limited dynamic range and low throughput. Similarly, RT-qPCR has high accuracy but cannot achieve whole transcriptome scale analyses[5–9]. RNA-seq has surpassed microarrays in both accuracy and dynamic range [10,11]. In a single cell, gene expression is intrinsically stochastic and cannot be synchronized among cells, which leads to cell-to-cell variations in mRNA expression levels[2,4,12]. This necessitates single cell transcriptome measurements, which have prompted intense recent efforts.

The first single-cell RNA-Seq method[10,11,13,14] was developed with PCR-based exponential amplification scheme, taking advantage of adding a poly-A tail to the 3’end of first-strand cDNAs by terminal transferase prior to the second strand synthesis. This PCR-based RNA-seq method lacked spike-in controls and displayed general amplification bias towards the 3’ ends of mRNAs as expected. Another PCR-based technique named Quartz-Seq[15] was developed with different strategy, while the same problems remained. Subsequent methods relied on a reverse transcriptase with template-switching activity, such as STRT[16–18] and SMART-seq[19–21]. Although they have the potential to amplify full-length mRNA, these PCR-based techniques may still consist of significant bias dependent on the length of mRNAs, considering the general preferences of PCR towards shorter amplicons. CEL-seq[22] and MARS-Seq[23] utilize in-vitro transcription (IVT) as the amplification method instead of PCR, and reduce hands-on time with the ability to pool many samples before amplification. At the same time, the requirement for barcoding limits coverage to only the 3’ or 5’ ends of the transcripts. Another method[24] based on random priming has been demonstrated recently, but could not address the low amplification efficiency issue.

Multiple annealing and looping-based amplification cycles (MALBAC)[25] was able to significantly reduce the amplification bias compared to previous MDA-based whole genome amplification[26]. It can also confidently detect copy-number variations and point mutations in the genome, presenting great downstream opportunities, such as profiling meiotic recombination and genome aneuploidy in sperm[27]. Taking advantage of its effectiveness in DNA amplification, here we present a single-cell transcriptome amplification method based on MALBAC, named MALBAC-RNA. Throughout this work, we systematically analyze the efficiency and technical consistency of this novel technique, and demonstrate its ability by applying it to single embryonic stem cells during mouse gastrulation.

Every organ or somatic tissue of a mouse is derived from a single sheet of epiblast[28,29]. During the gastrulation stage from 6.5 to 8.5 days *post coitum* (d.p.c.), the cup-shaped epiblast diversifies to generate three distinct germ layers known as ectoderm, mesoderm and endoderm. During this period, the mesoderm and endoderm delaminate from the epiblast in a specialized region, namely the primitive streak, which contains a narrow stripe of egressing and differentiating cells running down one side of the cup. Each layer then gives rise to different components of the fetal organ primordia. Therefore, gastrulation represents a crucial phase of cytodifferentiation, morphogenesis and pattern formation, dramatically transforming an epithelial sheet into an embryo with recognizable vertebrate form within 48 hours.

During the early stage of gastrulation, in order to move into the primitive streak in the embryo and further differentiate into 3 distinct germ layers, the epiblast cells have to lose their cell-cell adhesion through an epithelial-mesenchymal transition (EMT)[30]. With the induction of EMT, cells within the newly formed mesoderm layer acquire the characteristics of the mesenchymal cells[31].

Transcriptome profiling of each of the germ layers could shed light on the differences in gene expression between the ectoderm, mesoderm and visceral endoderm. However, the study of post-implantation embryonic development has been hampered by the limited amount of RNA obtainable from a mammalian embryo. Taking advantage of our MALBAC-RNA single cell sequencing method, we were able to compare single-cell transcriptomes between germ layers, which enables us to have a detailed look at the transcriptional network active during the EMT process.

## Materials and Methods

### Mouse embryo dissection

At 7.0 days post coitum (dpc), C57BL/6 mice were sacrificed under anesthesia by isoflurane overdose followed by cervical dislocation, and the embryos were collected. The extra embryonic tissues were mechanically removed in M2 medium with 10% fetal calf serum. The remaining embryonic region was rinsed in PBS and then digested with dispase, followed by mechanical dissection. The isolated ectoderm, mesoderm, visceral endoderm pieces were trypsinized into single cells, which were individually mouth picked into cell lysis buffer in PCR tubes for single-cell amplification. Animal experiments were approved by the Institutional Animal Care and Use Committees (IACUC) at Harvard University.

### Cell culture and sample preparation before single cell amplification

Obtained from American Type Culture Collection (ATCC), SW480 cells were cultured in Leibovitz’s L-15 Medium with 10% fetal bovine serum, 100 I.U./ml Penicillin and 100 μg/ml Streptomycin. Prior to the experiment, the cells were treated with 0.25% Trypsin-EDTA and washed once with 1x phosphate buffered saline (PBS). After the wash, cells are diluted and counted under the microscope to estimate the cell concentration. With calculated amount of dilution from the original cell suspension with 1xPBS, a final concentration of 100 cells/uL is reached. 1uL of the well-mixed diluted cell suspension is added into a total of 4uL cell lysis buffer, which contains 1x first-strand buffer for Superscript III Reverse Transcriptase, 5mM DTT, 0.5mM each dNTP mix, 0.45% IGEPAL CA-630, 0.4U/uL RNase inhibitor, 0.2U/uL SUPERase In, 2.5uM GAT-12dT primer. Cell is lysed by heating at 70°C for 90 seconds and then the reaction undergoes MALBAC-RNA amplification as described below.

### Multiple annealing and looping-based amplification cycles RNA amplification

To start reverse transcription, 0.33uL of superscript III reverse transcriptase, 0.07uL of T4 gene 32 protein, and 0.05uL of RNase inhibitor is added to the separated supernatant, then followed by a thermal cycling program with 4°C 2 minutes, 10°C 2 minutes, 20°C 2 minutes, 30°C 2 minutes, 40°C 2 minutes, 50°C 50 minutes and 70°C 15 minutes. Starting a similar MALBAC step, a 16uL reaction is mixed with the final concentration of 0.5uM GAT-7N primer, 1x thermo buffer, 0.4mM each dNTP mix, 1mM MgSO_4_ and 0.06U/uL deep vent (exo-) DNA Polymerase. MALBAC amplification starts with 95°C 5 minutes, and then 10 cycles of 20°C 50 seconds, 30°C 50 seconds, 40°C 45 seconds, 50°C 45 seconds, 65°C 4 minutes, 95°C 20 seconds, 58°C 20 seconds. After pre-amplification, a 14uL PCR mix, containing 0.36uM GAT-COM primer, 0.4uM each dNTP mix, 1x thermo buffer, 1uM MgSO_4_ and 0.06U/uL deep vent (exo-) DNA Polymerase, is added to the 16uL reaction from previous step. The PCR program starts with 95°C 1 minute, 19 cycles of 95°C 20 seconds, 58°C 30 seconds, 72°C 3 minutes, and a final 5 minutes additional extension at 72°C. Amplified cDNA products are purified with Zymo DNA Clean & Concentrator-5 and eluted into 50uL EB buffer. These MALBAC amplified DNA products are directly used for standard Illumina HiSeq library preparation.

### Library preparation and sequencing

For each sample, several micrograms of amplified cDNA were generated by the PCR amplification, following MALBAC-RNA. With the validation on a few housekeeping and highly expressed genes with qPCR, including Gapdh, Rps13, Rpl21, Rps8, Actb, libraries were constructed for Illumina HiSeq 2000 sequencer, with about 1ug cDNA from each sample. The number of reads for each cell sequenced ranges from 3 to 7 million, with 100 bp paired-end sequencing. All data are accessible at the NCBI Sequence Read Archive through the accession number SRP049515.

### Sequencing data analysis

Reads were aligned to the reference genome using Tophat 2.0.4[32] and FPKM values were estimated using Cufflinks 2.0.1[33]. Data from SW480 cells were aligned to genes annotated in the UCSC knownGenes table for the hg19 reference genome. Gene expression estimates were rescaled using upper quartile normalization of genes with detectable expression in at least one of the replicates[34]. For technical evaluations, spike-ins were limited to those with GC content between 40% and 60%. Data from mouse embryos and was aligned to the mm9 reference genome using RefSeq annotations. Hierarchical clustering was performed in R using heatmap.2 and differential expression analysis was performed using DESeq[35]. Gene ontology enrichment was performed using GOrilla[36].

### Primer sequences

#### GAT-12dT

5- GTG AGT GAT GGT TGA GGT AGT GTG GAG TTT TTT TTT TTT -3

#### GAT-7N

5- GTG AGT GAT GGT TGA GGT AGT GTG GAG NNN NNN N -3

#### GAT-COM

5- GTG AGT GAT GGT TGA GGT AGT GTG GAG -3

### Reagents list

M2 medium (Sigma-Aldrich, cat. no. M7167-100ML)
Fetal calf serum (Fisher Scientific, cat. no. R92157)
Phosphate buffered saline (PBS), 1x (Thermo Scientific, cat. no. SH30256.01)
Dispase (BD Biosciences, cat. no. 354235)
Leibovitz’s L-15 medium (ATCC, cat. no. 30–2008)
Fetal bovine serum (ATCC, cat. no. 30–2020)
Penicillin-Streptomycin, 100x (Mediatech, Inc., cat. no. 30-001-CI)
Trypsin-EDTA, 0.25% (Mediatech, Inc., cat. no. 25-053-CI)
Nuclease-free water (Ambion, cat. no. AM9937)
Dithiothreitol (DTT), 1M (Life Technologies, cat. no. P2325)
Superscript III Reverse Transcriptase (Life Technologies, cat. no. 18080–044)
First-strand buffer, 5x (Life Technologies, cat. no. 18080–044)
dNTP Mix, 10mM each (New England Bioloabs, Inc., cat. no. N0447L)
IGEPAL CA-630 (Sigma-Aldrich, cat. no. I8896-50ML)
RNase inhibitor (40U/μL) (Life Technologies, cat. no. AM2682)
SUPERase-In (20U/μL) (Life Technologies, cat. no. cat. no. AM2694)
T4 gene 32 protein (New England Bioloabs, Inc., cat. no. M0300L)
ThermoPol reaction buffer, 10x (New England Bioloabs, Inc., cat. no. B9004S)
Magnesium Sulfate (MgSO_4_) Solution (100 mM) (New England Bioloabs, Inc., cat. no. B1003S)
Deep-ventR (exo-) DNA Polymerase (2,000 U/mL) (New England Bioloabs, Inc., cat. no. M0259L)
DNA clean & concentrator-5 (Zymo Research, cat. no. D4013)

## Results and Discussion

### Single cell transcriptome amplification with MALBAC-RNA

**Fig 1:**
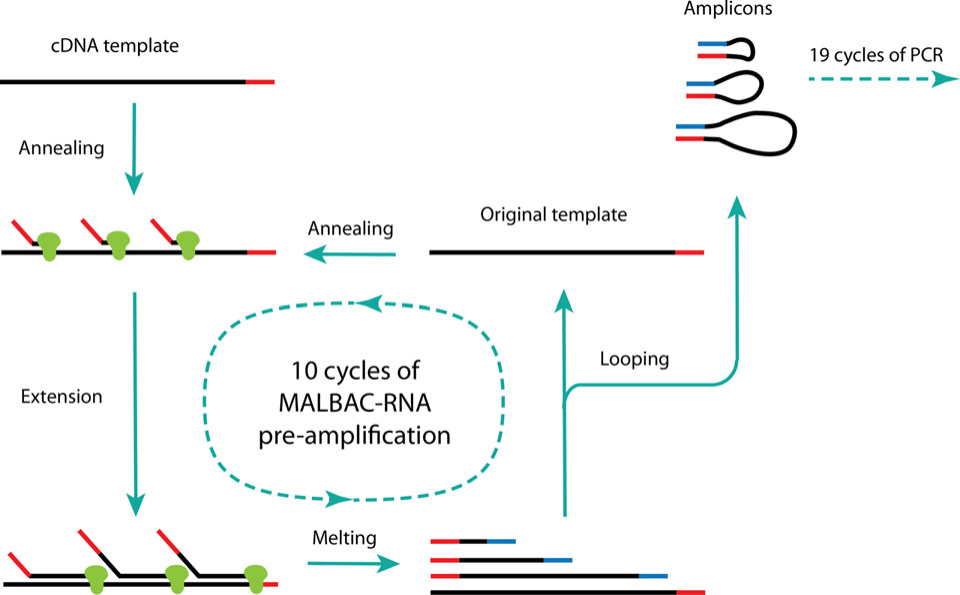
Single-cell MALBAC-RNA amplification diagram. After reverse transcription, primers with 7 random nucleotides at the 3’ end are annealed to the cDNA template at 4°C, then extended by DNA polymerase with strand displacement activity as temperature is increased. Amplicons are then melted off the original template after DNA extension, and looped at 58°C to protect themselves from being further amplified thanks to their 5’ ends being complementary to their 3’ ends. This MALBAC-RNA step includes a total of 10 cycles of quasilinear amplification, followed by another 19 cycles of PCR.

During the experiment, each cell is picked and transferred into PCR reaction tubes preloaded with mild cell lysis buffer. After cell lysis, mRNA is reverse transcribed to cDNA with poly-T primers, which include a 27-nucleotide sequence. With cDNA being synthesized, the same 27 nucleotides together with 7 random nucleotides are used for cDNA amplification. Those 7 random nucleotides can hybridize evenly onto reverse transcribed cDNA at 4°C (Fig 1). As the temperature is slowly increased to 65°C, second strand cDNA synthesis is started. With strand displacement activity, DNA polymerase enables the primer from behind to displace the primer downstream base-by-base as it proceeds along the template. Upon reaching the end of extension, each newly synthesized cDNA has a 27-base tag at its 3’ end complementary to its 5’ end, thanks to the same sequence being used at both reverse transcription and second strand synthesis. In order to avoid being further amplified, after being melted at 95°C, cDNA with complementary tags at both ends is able to form a loop at 58°C, finishing a full MALBAC cycle. A total of 10 MALBAC pre-amplification cycles are used to generate enough amplicons for PCR. Since during each cycle, only the original cDNA template is targeted for amplification, MALBAC does not generate as much bias, and the overall amplification efficiency is quasilinear. In order to acquire enough material for sequencing, a further 19-cycle PCR amplification is applied using the same 27-base common sequence as in the primers.

**Fig 2:**
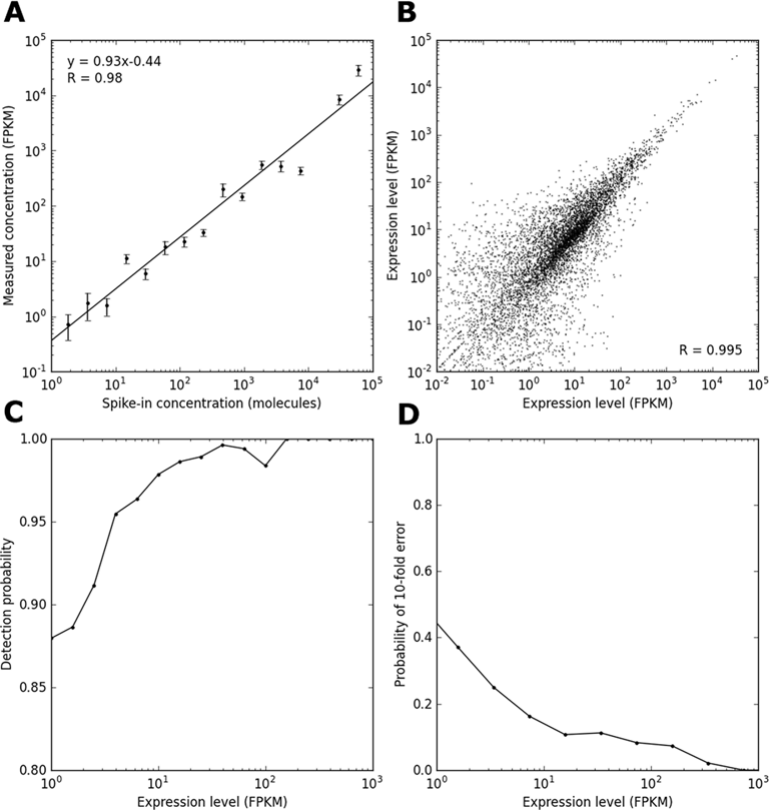
Technical reproducibility of MALBAC-RNA amplification. (A) Mean expression level measured in across two technical replicates and nine SW480 single cells for synthetic spike-ins of a particular concentration. Error bars represent standard errors. (B) Scatter plot of two technical replicates exhibits a high correlation coefficient (R = 0.995). To prepare technical replicates, single-cell amount of RNA was aliquoted from 100 cells after cell membrane lysis and they should only differ by Poisson fluctuations in molecular counts. (C) Probability of detecting a transcript in one technical replicate as a function of its expression level in the other replicate. (C) Probability that the expression level of a transcript in one replicate will differ by at least 10-fold from the measurement in the other replicate.

To evaluate the technical reproducibility of MALBAC-RNA, we amplified two replicates by diluting and aliquoting a 100-cell lysate from the colorectal cancer cell line SW480 into single-cell portions. These technical replicates should differ in molecular counts only by Poisson fluctuations. Additionally, we amplified and sequenced nine SW480 single cells which would exhibit biological variability as well. MALBAC-RNA exhibits a linear detection of synthetic spike-in transcripts across five orders of magnitude (Fig 2A). Of the 11,233 genes detected in bulk mRNA sequencing, only 1045 were not detected in at least one of the single cells, while an additional 1622 genes were detected in at least one of the single cells but not the bulk. The correlation between the two technical replicates is shown in Fig 2B. MALBAC-RNA shows high reproducibility with a correlation coefficient of 0.995, while the nine SW480 single cells exhibited reduced correlation due to biological variations between cells (Figure A in S1 File). However, correlation is primarily influenced by a handful of highly expressed genes and is therefore a poor metric for evaluating technical reproducibility (Figure B in S1 File). More tellingly, MALBAC-RNA exhibits reproducibility in detecting expressed genes (Fig 2C) with low amplification noise, as depicted by a 10-fold or more FPKM difference for the same gene after amplification (Fig 2D). Moreover, because random primers are incorporated throughout the transcripts, amplification is not biased against longer transcripts (Figure C in S1 File).

### Transcriptome Amplification of Single Embryonic Stem Cells

Having demonstrated that MALBAC-RNA generates quantitative and reproducible single-cell transcriptomes, we asked whether a global analysis of cells isolated from early gastrulation stage embryos could reveal germ layer-specific transcriptomic patterns and trace the origin of germ-layer derivation.

**Fig 3:**
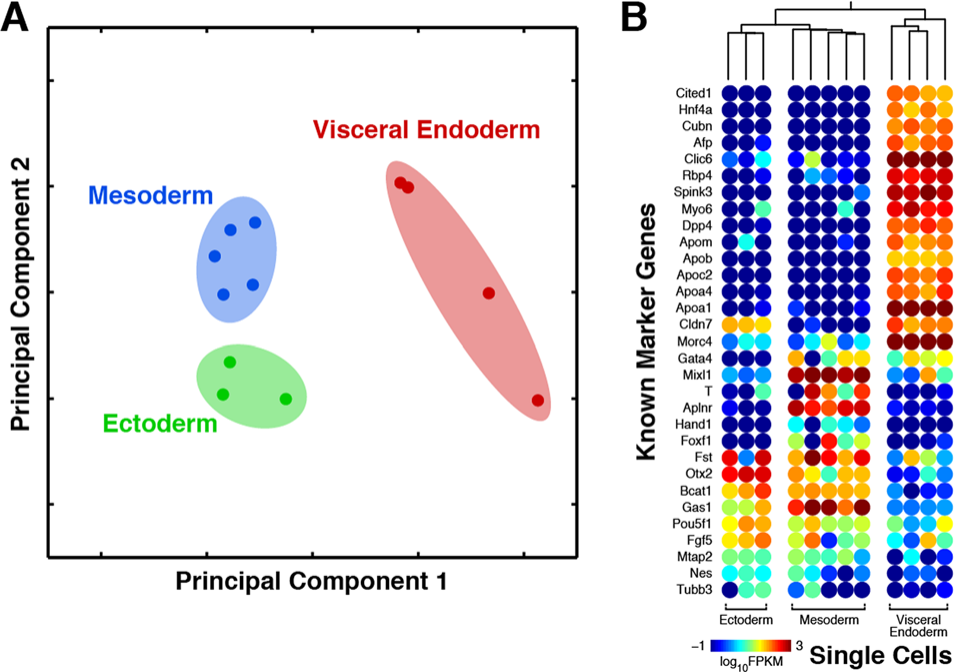
Gene expression profiles of 7.0dpc mouse embryo stem cells from 3 different germ layers. MALBAC-RNA distinguishes single cells from different germ layers of a post-implantation mouse embryo (7.0dpc). A total of 12 single cells were isolated from a 7.0dpc mouse embryo, among which 3 were from the ectoderm, 5 from the mesoderm, and 4 from the visceral endoderm. (A) Principle component analysis of transcriptomes clearly separates the 12 single cells into three clusters, each representing one germ layer. (B) Top: Hierarchical clustering of transcriptomes classifies the 12 single cells into three non-overlapping sub-trees representing the three germ layers. Bottom: Known marker genes of the three germ layers exhibit strong layer-specific patterns of expression, although some show significant cell-to-cell variation within a layer. Principle component analysis and hierarchical clustering were based on the ranking of each gene’s FPKM among all cells.

To this end, we amplified and sequenced a total of 11 single-cell transcriptomes from each of the three germ layers—ectoderm, mesoderm, and visceral endoderm—from a 7.0dpc embryo (S1 Table). With principal component analysis, samples from the three germ layers were clearly separated (Fig 3A). In particular, the first principal component distinguishes the visceral endoderm from the other two layers, whereas the second principal component represents the difference between ectoderm and mesoderm. The germ-layer origin of these embryonic cells is additionally confirmed by the expression of known germ-layer-specific markers (Fig 3B). All visceral endoderm cells express high levels for endoderm specific marker genes, such as Cited1, Hnf4a, Cubn, Afp, Apoa1, but not mesoderm markers, such as Aplnr. The data show a distinct differentiation for the 3 germ layers based on their unique expression profiles. A total of 738 genes were found to be differentially expressed between ectoderm and mesoderm, 1783 between ectoderm and visceral endoderm, and 1831 between mesoderm and visceral endoderm. Differentially expressed genes were enriched for processes including those related to embryonic morphogenesis, pattern specification processes, cell differentiation, and regulation of Wnt signaling pathway (S3 Table). Therefore, both the global transcriptomes and the expression of known germ layer associated marker genes clearly support the germ-layer identity of all the examined single cells.

We next investigated the relationship between the three germ layers. Interestingly, they are not equally separated from each other. In principal component analysis, the visceral endoderm is more distinct from the other two layers and this difference constitutes the most significant component of variance. Consistent with this observation, hierarchical clustering placed ectoderm and mesoderm under the same subtree (Fig 3B). Therefore, our data suggest a more distinct separation of visceral endoderm from the other two germ layers at 7.0dpc.

**Fig 4:**
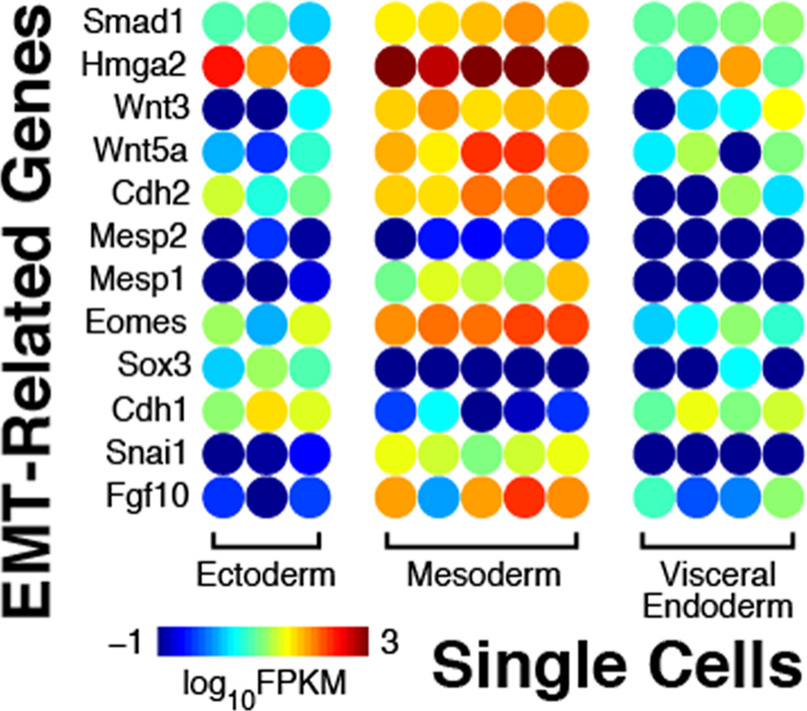
Gene expression heat map of EMT-related genes. Genes related to epithelial-mesenchymal transition (EMT) are differentially expressed across the three germ layers. Among them, FGF10 and Snai1 are significantly overexpressed in the mesoderm, whereas E-cadherin and Sox3 are depleted. At the same time, Eomes and Mesp1 are highly expressed in the mesoderm, although Mesp2 is not significantly expressed. Other EMT-related genes, including CDH2, Wnt5a, Wnt3, Hmga2, Smad1, and Fgf10, are also enriched in the mesoderm, which confirms the cellular transitions during gastrulation.

In addition, we also investigated the results of EMT programming within the mesoderm, as compared to the other two germ layers, based on their single-cell transcriptomes. As can be seen in Fig 4, both FGF10 and Snai1 have been significantly overexpressed in mesoderm, whereas the E-cadherin level is lowered compared to ectoderm and visceral endoderm, indicating the downregulation of E-cadherin expression by FGF signals, through the regulation of *snail* gene expression[37]. As Sox3 genes have been completely depleted in mesoderm, the reciprocal repression between Snail and Sox3 is suggested in our experiment as well as previously reported[38]. At the same time, both Eomes and Mesp1 are highly expressed in the mesoderm, supporting the theory that Eomes acts upstream of Mesp[30], although in our data only the upregulation of Mesp1 is observed rather than both Mesp1 and Mesp2. A few other EMT signature genes have also been found significantly overexpressed in mesoderm cells, like CDH2, Wnt5a, Wnt3, Hmga2, Smad1, Fgf10, which further confirms the transition of the cells in gastrulation stage.

Lastly, MALBAC-RNA revealed novel patterns of gene expression during early gastrulation. Some known germ-layer-specific markers for mesoderm, like BMP2, are not expressed in our samples. In some cases a known marker, such as T, is observed but only in one or two of the corresponding single cells. In addition, we identified new genes that are specific for each germ layer. For example Cotl1, a Coactosin-like protein, is found to be highly expressed in all cells from visceral endoderm.

## Conclusions

In this work, we developed a new single-cell transcriptome amplification method based on MALBAC. Instead of performing a second strand synthesis right after the reverse transcription, as is usually done by most other RNA amplification methods, we deployed a modified version of MALBAC genome amplification on first-strand synthesized cDNA directly, followed by PCR amplification. Furthermore, we showed that MALBAC-RNA has great amplification sensitivity and consistency, especially for the genes at relatively low expression levels.

Although a critical stage during embryonic development, gastrulation has never been thoroughly studied transcriptome-wide on a single-cell level. And recently, there has been a strong interest in identifying the key components in the transcriptomes of different germ layers during gastrulation. To demonstrate our ability to amplify single-cell transcriptomes with MALBAC-RNA, the complete transcriptomes for the three germ layers of early gastrulation in mouse are uncovered for the first time at single-cell and single-base resolution.

With the availability of the single-cell transcriptomes from the early gastrulation stage of mouse embryos, we were able to examine the EMT process during embryonic development. We successfully found some of the transcriptional networks on single-cell levels as suggested in previous research. The cells from the mesoderm layer showed characteristics of the cells that went through EMT, compared to the other two germ layers that were sequenced. This analysis demonstrates that as an accurate single-cell RNA amplification method, MALBAC-RNA could be used to analyze certain cellular mechanisms on a single-cell level, which provides more detailed information than would be possible with bulk population analysis.

Probing gene expression in small populations of cells *in vivo* is critical for the study of developmental biology. Although cell lines exist to imitate some of these processes *in vitro*, which provide a large amount of RNA for molecular analysis, many events that involve complex morphogenesis and pattern formation, such as the mammalian gastrulation, can only be studied in living embryos. These cases call for a reliable technique that directly assesses changes in gene expression *in vivo*. In particular, this technique should not be limited to studying genes that are already identified due to their activity in other biological systems, because such approaches impose an inherent prejudice and may thus overlook novel pathways or responses. Here, we precisely micro-dissected single cells form an embryo in its gastrulation stage and sequenced the transcriptomes of three to five individual cells from each of the three germ layers. This provides a useful resource for studying differential gene expression between the three germ layers—the three most important cell populations within gastrulating embryos. The validity of the micro-dissection was confirmed by unsupervised hierarchical clustering, correlation analyses of gene expression levels and the profiling of known marker genes from each germ layer. We find that single cells from the same germ layer exhibit similar gene expression patterns. Therefore, we demonstrate that analyses of germ-layer-specific gene expression can provide a rapid screen for novel genes that are expressed in a tissue- or region-specific manner.

## Supporting Information

S1 File Figure A: Biological variation among single cells. Scatter plots showing gene expression levels (FPKM) between pairs of nine SW480 single cells, with their respective correlation coefficients in the lower half. **Figure B: Correlation is strongly influenced by highly expressed genes**. Scatter plots showing correlation coefficients of MALBAC technical replicates with (A) all genes included (99.5%), (B) the highest 0.1% of genes excluded (94.5%), and (C) the highest 1.0% of genes excluded (81.8%). The correlation as commonly reported can vary greatly due to the expression of relatively small number of house keeping genes or the amount of spike-ins added. **Figure C: Amplification biases for genes of varying lengths**. Genes were binned into 1kb buckets by length. For each gene, the bias was calculated as (μ-b)/(μ+b), where μ is the average FPKM among the two technical replicates and nine single cells, and b is the FPKM of the gene in the bulk sample. For each length bin, the average across all genes in the bin is presented.
(DOCX)

S1 Table Statistics of sequencing data. Number of reads sequenced and mapped for embryo cells. Three cells were sequenced from the ectoderm (EC1-3), five from the mesoderm (ME1-5) and four from the visceral endoderm (VE1-4).
(DOCX)

S2 Table Gene expression levels in embryo cells. FPKM values are listed for all genes in each of the 11 embryo cells sequenced.
(XLSX)

S3 Table Enrichment of gene ontology terms for differentially expressed genes. Enriched gene ontology terms, along with corresponding fold-enrichment and uncorrected and multiple testing corrected p-values, are listed for genes differentially expressed across each pair of germ layers.
(XLSX)

## References

[1] Mortazavi A, Williams BA, McCue K, Schaeffer L, Wold B. Mapping and quantifying mammalian transcriptomes by RNA-Seq. Nat Meth. 2008;5: 621–628. 10.1038/nmeth.1226 PMC13303166 18516045

[2] Raj A, Peskin CS, Tranchina D, Vargas DY, Tyagi S. Stochastic mRNA Synthesis in Mammalian Cells. PLoS Biol. 2006;4: e309 10.1371/journal.pbio.0040309 17048983 PMC1563489

[3] Topalidou I, Oudenaarden A van, Chalfie M. Caenorhabditis elegans aristaless/Arx gene alr-1 restricts variable gene expression. PNAS. 2011;108: 4063–4068. 10.1073/pnas.1101329108 21368126 PMC3053973

[4] Taniguchi Y, Choi PJ, Li G-W, Chen H, Babu M, Hearn J, et al Quantifying E. coli Proteome and Transcriptome with Single-Molecule Sensitivity in Single Cells. Science. 2010;329: 533–538. 10.1126/science.1188308 20671182 PMC2922915

[5] Warren LA, Rossi DJ, Schiebinger GR, Weissman IL, Kim SK, Quake SR. Transcriptional instability is not a universal attribute of aging. Aging Cell. 2007;6: 775–782. 10.1111/j.1474-9726.2007.00337.x 17925006

[6] Bengtsson M, Ståhlberg A, Rorsman P, Kubista M. Gene expression profiling in single cells from the pancreatic islets of Langerhans reveals lognormal distribution of mRNA levels. Genome Res. 2005;15: 1388–1392. 10.1101/gr.3820805 16204192 PMC1240081

[7] Diehn M, Cho RW, Lobo NA, Kalisky T, Dorie MJ, Kulp AN, et al Association of reactive oxygen species levels and radioresistance in cancer stem cells. Nature. 2009;458: 780–783. 10.1038/nature07733 19194462 PMC2778612

[8] Sanchez-Freire V, Ebert AD, Kalisky T, Quake SR, Wu JC. Microfluidic single-cell real-time PCR for comparative analysis of gene expression patterns. Nat Protocols. 2012;7: 829–838. 10.1038/nprot.2012.021 22481529 PMC3657501

[9] Guo G, Huss M, Tong GQ, Wang C, Li Sun L, Clarke ND, et al Resolution of Cell Fate Decisions Revealed by Single-Cell Gene Expression Analysis from Zygote to Blastocyst. Developmental Cell. 2010;18: 675–685. 10.1016/j.devcel.2010.02.012 20412781

[10] Tang F, Barbacioru C, Wang Y, Nordman E, Lee C, Xu N, et al mRNA-Seq whole-transcriptome analysis of a single cell. Nature Methods. 2009;6: 377–382. 10.1038/nmeth.1315 19349980

[11] Tang F, Barbacioru C, Nordman E, Li B, Xu N, Bashkirov VI, et al RNA-Seq analysis to capture the transcriptome landscape of a single cell. Nature Protocols. 2010;5: 516–535. 10.1038/nprot.2009.236 20203668 PMC3847604

[12] Raser JM, O’Shea EK. Control of stochasticity in eukaryotic gene expression. Science. 2004;304: 1811–1814. 10.1126/science.1098641 15166317 PMC1410811

[13] Tang F, Lao K, Surani MA. Development and applications of single-cell transcriptome analysis. Nature Methods. 2011; 10.1038/nmeth.1557 PMC3408593 21451510

[14] Yan L, Yang M, Guo H, Yang L, Wu J, Li R, et al Single-cell RNA-Seq profiling of human preimplantation embryos and embryonic stem cells. Nat Struct Mol Biol. 2013;20: 1131–1139. 10.1038/nsmb.2660 23934149

[15] Sasagawa Y, Nikaido I, Hayashi T, Danno H, Uno KD, Imai T, et al Quartz-Seq: a highly reproducible and sensitive single-cell RNA sequencing method, reveals non-genetic gene-expression heterogeneity. Genome Biology. 2013;14: R31 10.1186/gb-2013-14-4-r31 23594475 PMC4054835

[16] Islam S, Kjällquist U, Moliner A, Zajac P, Fan J-B, Lönnerberg P, et al Characterization of the single-cell transcriptional landscape by highly multiplex RNA-seq. Genome Res. 2011;21: 1160–1167. 10.1101/gr.110882.110 21543516 PMC3129258

[17] Islam S, Kjällquist U, Moliner A, Zajac P, Fan J-B, Lönnerberg P, et al Highly multiplexed and strand-specific single-cell RNA 5′ end sequencing. Nat Protocols. 2012;7: 813–828. 10.1038/nprot.2012.022 22481528

[18] Islam S, Zeisel A, Joost S, La Manno G, Zajac P, Kasper M, et al Quantitative single-cell RNA-seq with unique molecular identifiers. Nat Meth. 2014;11: 163–166. 10.1038/nmeth.2772 24363023

[19] Picelli S, Björklund ÅK, Faridani OR, Sagasser S, Winberg G, Sandberg R. Smart-seq2 for sensitive full-length transcriptome profiling in single cells. Nat Meth. 2013;10: 1096–1098. 10.1038/nmeth.2639 24056875

[20] Picelli S, Faridani OR, Björklund ÅK, Winberg G, Sagasser S, Sandberg R. Full-length RNA-seq from single cells using Smart-seq2. Nat Protocols. 2014;9: 171–181. 10.1038/nprot.2014.006 24385147

[21] Ramsköld D, Luo S, Wang Y-C, Li R, Deng Q, Faridani OR, et al Full-length mRNA-Seq from single-cell levels of RNA and individual circulating tumor cells. Nature Biotechnology. 2012;30: 777–782. 10.1038/nbt.2282 22820318 PMC3467340

[22] Hashimshony T, Wagner F, Sher N, Yanai I. CEL-Seq: Single-Cell RNA-Seq by Multiplexed Linear Amplification. Cell Reports. 2012;2: 666–673. 10.1016/j.celrep.2012.08.003 22939981

[23] Jaitin DA, Kenigsberg E, Keren-Shaul H, Elefant N, Paul F, Zaretsky I, et al Massively Parallel Single-Cell RNA-Seq for Marker-Free Decomposition of Tissues into Cell Types. Science. 2014;343: 776–779. 10.1126/science.1247651 24531970 PMC4412462

[24] Pan X, Durrett RE, Zhu H, Tanaka Y, Li Y, Zi X, et al Two methods for full-length RNA sequencing for low quantities of cells and single cells. PNAS. 2013;110: 594–599. 10.1073/pnas.1217322109 23267071 PMC3545756

[25] Zong C, Lu S, Chapman AR, Xie XS. Genome-Wide Detection of Single-Nucleotide and Copy-Number Variations of a Single Human Cell. Science. 2012;338: 1622–1626. 10.1126/science.1229164 23258894 PMC3600412

[26] Dean FB, Nelson JR, Giesler TL, Lasken RS. Rapid Amplification of Plasmid and Phage DNA Using Phi29 DNA Polymerase and Multiply-Primed Rolling Circle Amplification. Genome Res. 2001;11: 1095–1099. 10.1101/gr.180501 11381035 PMC311129

[27] Lu S, Zong C, Fan W, Yang M, Li J, Chapman AR, et al Probing Meiotic Recombination and Aneuploidy of Single Sperm Cells by Whole-Genome Sequencing. Science. 2012;338: 1627–1630. 10.1126/science.1229112 23258895 PMC3590491

[28] Beddington RS., Robertson EJ. Anterior patterning in mouse. Trends in Genetics. 1998;14: 277–284. 10.1016/S0168-9525(98)01499-1 9676530

[29] Beddington RS., Robertson EJ. Axis Development and Early Asymmetry in Mammals. Cell. 1999;96: 195–209. 10.1016/S0092-8674(00)80560-7 9988215

[30] Lim J, Thiery JP. Epithelial-mesenchymal transitions: insights from. Development. 2012;139: 3471–3486. 10.1242/dev.071209 22949611

[31] Nakaya Y, Sheng G. Epithelial to mesenchymal transition during gastrulation: An embryological view. Development, Growth & Differentiation. 2008;50: 755–766. 10.1111/j.1440-169X.2008.01070.x 19046163

[32] Kim D, Pertea G, Trapnell C, Pimentel H, Kelley R, Salzberg SL. TopHat2: accurate alignment of transcriptomes in the presence of insertions, deletions and gene fusions. Genome Biology. 2013;14: R36 10.1186/gb-2013-14-4-r36 23618408 PMC4053844

[33] Trapnell C, Hendrickson DG, Sauvageau M, Goff L, Rinn JL, Pachter L. Differential analysis of gene regulation at transcript resolution with RNA-seq. Nat Biotech. 2013;31: 46–53. 10.1038/nbt.2450 PMC3869392 23222703

[34] Bullard JH, Purdom E, Hansen KD, Dudoit S. Evaluation of statistical methods for normalization and differential expression in mRNA-Seq experiments. BMC Bioinformatics. 2010;11: 94 10.1186/1471-2105-11-94 20167110 PMC2838869

[35] Anders S, Huber W. Differential expression analysis for sequence count data. Genome Biology. 2010;11: R106 10.1186/gb-2010-11-10-r106 20979621 PMC3218662

[36] Eden E, Navon R, Steinfeld I, Lipson D, Yakhini Z. GOrilla: a tool for discovery and visualization of enriched GO terms in ranked gene lists. BMC Bioinformatics. 2009;10: 48 10.1186/1471-2105-10-48 19192299 PMC2644678

[37] Ciruna B, Rossant J. FGF Signaling Regulates Mesoderm Cell Fate Specification and Morphogenetic Movement at the Primitive Streak. Developmental Cell. 2001;1: 37–49. 10.1016/S1534-5807(01)00017-X 11703922

[38] Acloque H, Ocaña OH, Matheu A, Rizzoti K, Wise C, Lovell-Badge R, et al Reciprocal Repression between Sox3 and Snail Transcription Factors Defines Embryonic Territories at Gastrulation. Developmental Cell. 2011;21: 546–558. 10.1016/j.devcel.2011.07.005 21920318 PMC3256632

