# Supplementary material for: Single Cell Transcriptome Amplification with MALBAC
Source: PLoS One. 2015 Mar 30;10(3):e0120889. doi: 10.1371/journal.pone.0120889 (PMC4378937; doi:10.1371/journal.pone.0120889)
Supplement: S1 File — Scatter plots showing gene expression levels (FPKM) between pairs of nine SW480 single cells, with their respective correlation coefficients in the lower half. Figure B: Correlation is strongly influenced by highly expressed genes. Scatter plots showing correlation coefficients of MALBAC technical replicates with (A) all genes included (99.5%), (B) the highest 0.1% of genes excluded (94.5%), and (C) the highest 1.0% of genes excluded (81.8%). The correlation as commonly reported can vary greatly due to the expression of relatively small number of house keeping genes or the amount of spike-ins added. Figure C: Amplification biases for genes of varying lengths. Genes were binned into 1kb buckets by length. For each gene, the bias was calculated as (μ-b)/(μ+b), where μ is the average FPKM among the two technical replicates and nine single cells, and b is the FPKM of the gene in the bulk sample. For each length bin, the average across all genes in the bin is presented. (DOCX) [file pone.0120889.s001.docx]

**Supporting Information Figures**


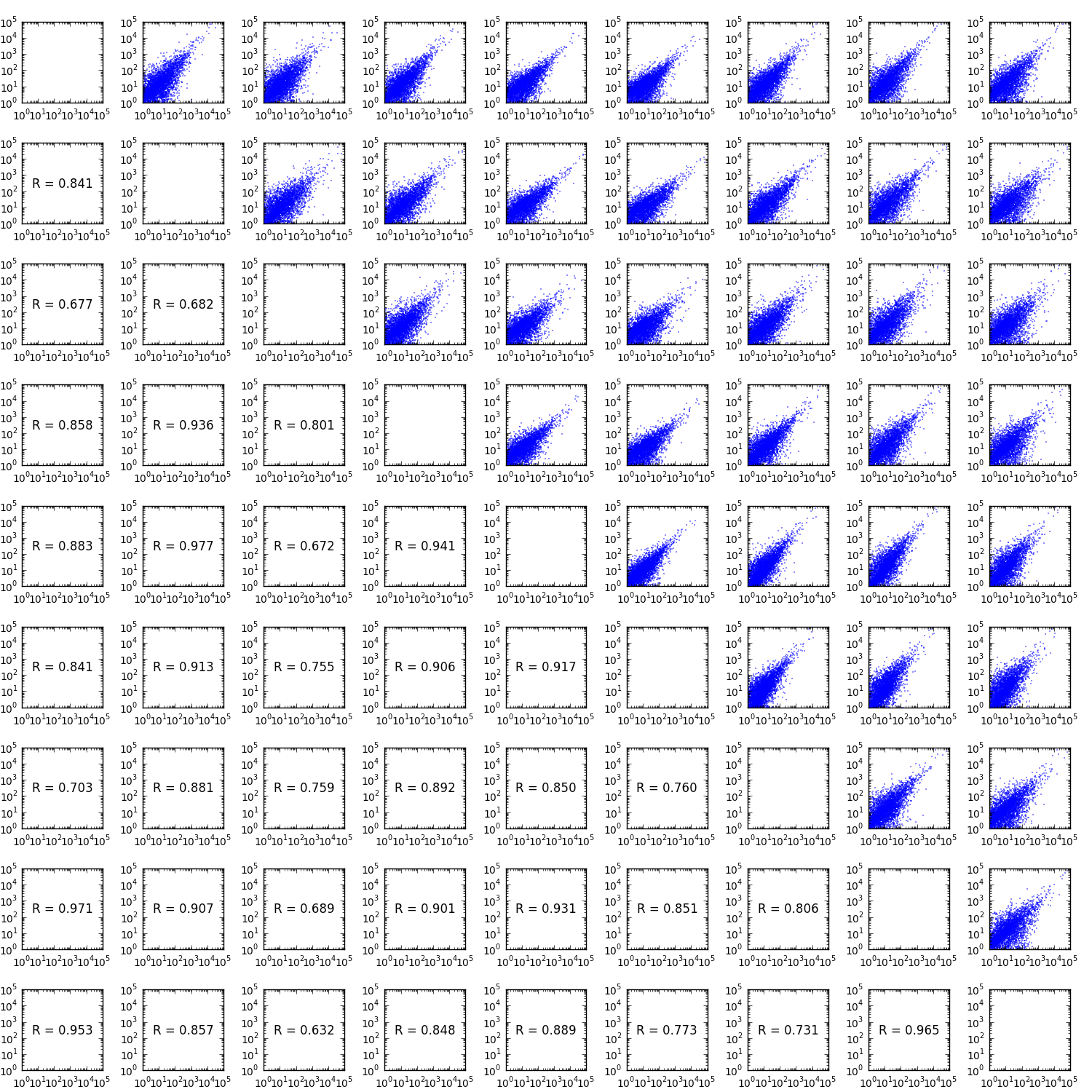


**Figure A– Biological variation among single cells**

Scatter plots showing gene expression levels (FPKM) between pairs of nine SW480 single cells, with their respective correlation coefficients in the lower half.

**
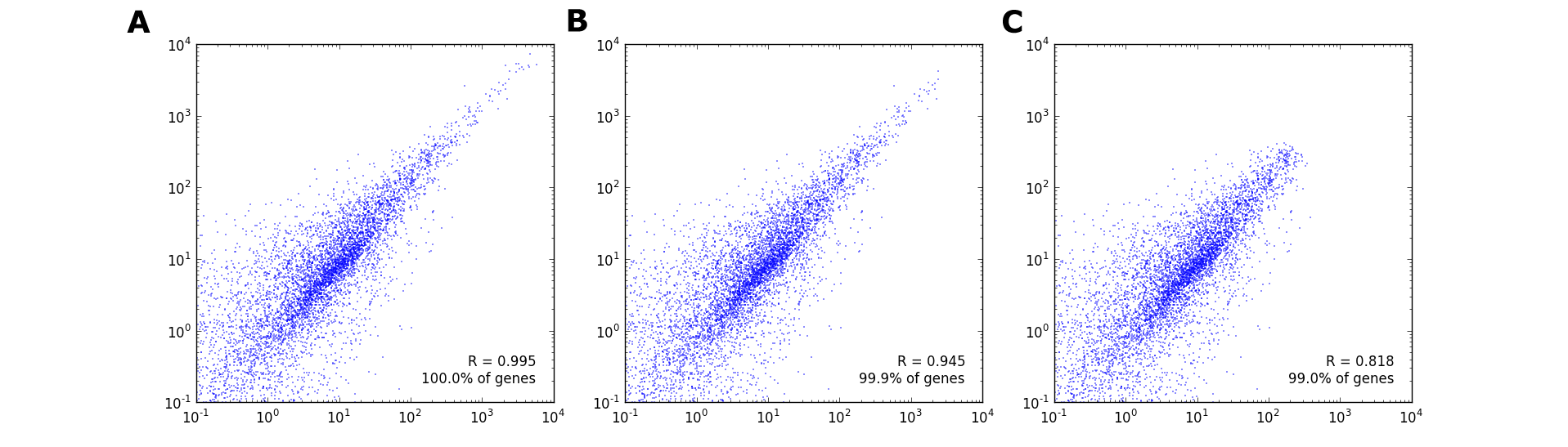
**

**Figure B – Correlation is strongly influenced by highly expressed genes**

Scatter plots showing correlation coefficients of MALBAC technical replicates with (A) all genes included (99.5%), (B) the highest 0.1% of genes excluded (94.5%), and (C) the highest 1.0% of genes excluded (81.8%). The correlation as commonly reported can vary greatly due to the expression of relatively small number of house keeping genes or the amount of spike-ins added.


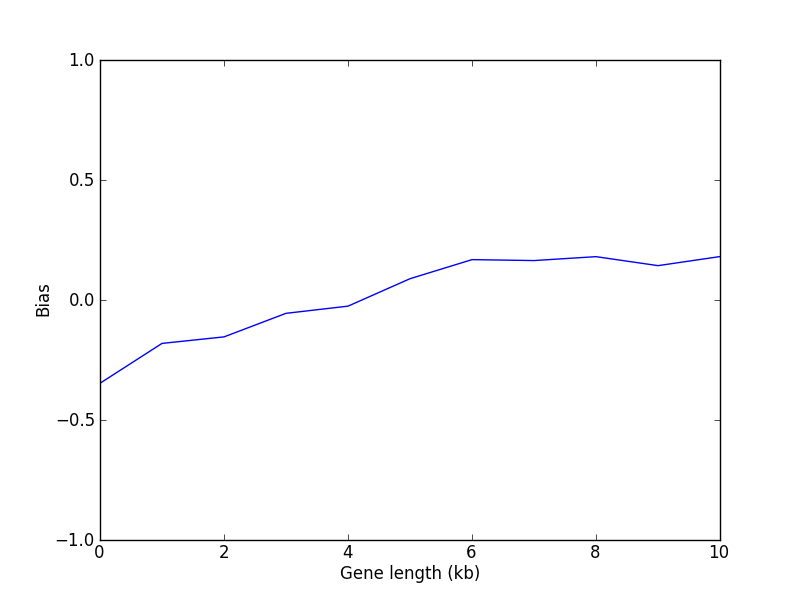


**Figure C – Amplification biases for genes of varying lengths**

Genes were binned into 1kb buckets by length. For each gene, the bias was calculated as (µ-b)/(µ+b), where µ is the average FPKM among the two technical replicates and nine single cells, and b is the FPKM of the gene in the bulk sample. For each length bin, the average across all genes in the bin is presented.
