# Supplementary material for: Single Cell Transcriptome Amplification with MALBAC
Source: PLoS One. 2015 Mar 30;10(3):e0120889. doi: 10.1371/journal.pone.0120889 (PMC4378937; doi:10.1371/journal.pone.0120889)
Supplement: S1 Table — Number of reads sequenced and mapped for embryo cells. Three cells were sequenced from the ectoderm (EC1-3), five from the mesoderm (ME1-5) and four from the visceral endoderm (VE1-4). (DOCX) [file pone.0120889.s002.docx]

| Sample | Number of read pairs | Number of reads mapped | Properly paired  (% of mapped reads) |
| --- | --- | --- | --- |
| EC1 | 4870310 | 6578033 | 64.3% |
| EC2 | 6699299 | 9730075 | 77.2% |
| EC3 | 6852313 | 8693994 | 73.0% |
| ME1 | 6818999 | 7558243 | 73.6% |
| ME2 | 6746402 | 8793896 | 74.8% |
| ME3 | 3888411 | 5285181 | 74.7% |
| ME4 | 6129738 | 6564571 | 75.1% |
| ME5 | 7239530 | 8041463 | 75.6% |
| VE1 | 4688183 | 4575148 | 68.5% |
| VE2 | 6257318 | 6765190 | 61.0% |
| VE3 | 5331697 | 5575674 | 64.9% |
| VE4 | 5929157 | 3946605 | 73.2% |

**Table S1 – Statistics of sequencing data**

Number of reads sequenced and mapped for embryo cells. Three cells were sequenced from the ectoderm (EC1-3), five from the mesoderm (ME1-5) and four from the visceral endoderm (VE1-4).
